# Hot and Cool Executive Functions in Adolescence: Development and Contributions to Important Developmental Outcomes

**DOI:** 10.3389/fpsyg.2017.02311

**Published:** 2018-01-10

**Authors:** Kean Poon

**Affiliations:** Department of Special Education and Counselling, Education University of Hong Kong, Tai Po, Hong Kong

**Keywords:** adolescence, executive functions, academic performance, psychological adjustment

## Abstract

Despite significant theoretical advancement in the area of child neuropsychology, limited attention has been paid to the developmental features of adolescence. The present study intends to address this issue in relation to executive function (EF). EF refers to the psychological processes that underlie goal-directed behavior; recent studies separate cool EF (psychological process involves pure logic and critical analysis) and hot EF (psychological process driven by emotion). Although neurological findings suggest that adolescence is a sensitive period for EF development, data on comparing the developmental progression in hot or cool EFs is highly missing. Moreover, while evidence has confirmed the relationships between EF and day-to-day functioning, whether and how hot and cool EFs contribute to core developmental outcomes in adolescence is still remained unknown. The current study aims to enhance our understanding of the development and impacts of hot and cool EFs in adolescence. A total of 136 typically developing adolescents from age 12 to 17 completed four cool EF tasks including Backward digit span, Contingency naming test, Stockings of Cambridge, and Stroop Color and Word test, and one hot task on Cambridge gambling task. Data on academic performance and psychological adjustment was also collected. Results showed that cool and hot EF exhibited different patterns of age-related growth in adolescence. Specifically, cool EF ascended with age while hot EF showed a bell-shaped development. Moreover, there were correlations among cool EF measures but no association between cool and hot EFs. Further, cool EF was a better predictor of academic performance, while hot EF uniquely related to emotional problems. The results provide evidence for the association among cool EF tests and the differentiation of hot and cool EFs. The bell-shaped development of hot EF might suggest a period of heightened risk-taking propensity in middle adolescence. Given the plastic nature of EF, especially over adolescence, the current findings may have practical implications for future EF identification and training.

## Introduction

### Background

Executive function (EF) is commonly considered an umbrella term, involving the skills essential for conscious, goal-directed thought and behavior (Shallice, 1982; Stuss and Benson, 1986; Miyake et al., 2000; Best and Miller, 2010; Diamond and Lee, 2011; Prencipe et al., 2011; Blair and Raver, 2012). Historically, EF has been understood through a purely cognitive lens, meaning the role of motivation and emotion in EF has been ignored. EF has been seen as purely cognitive skills that are elicited under relatively abstract, decontextualized, non-affective conditions (Peterson and Welsh, 2014). Research in the last decade has suggested that EF may operate differently in different contexts (e.g., Bechara, 2004), suggesting the importance of emotion regulation and bringing to light the “hot” affective aspects of EF (Zelazo and Cunningham, 2007; Zelazo et al., 2010). This broader conceptualization of EF has important implications for developmental research because EF has been found to be a strong predictor of school readiness, academic achievement and social behavior (Brock et al., 2009; Jacobson et al., 2011).

Although the composition of EF is debatable, it is generally agreed that core EFs are cognitive flexibility, inhibition, and working memory. More complex EFs include planning and organizing (Miyake et al., 2000). This traditional EF construct has been categorized as “cool” EF. Cool components required large amount logic and critical analysis (Rubia, 2011), and usually involve conscious control of thoughts and actions without an affective component. For instance, in the Wisconsin Card Sorting Test (WCST; Grant and Berg, 1948), widely considered to be “the prototypical EF task” (Pennington and Ozonoff, 1996, p. 55), participants are provided test cards that had three different dimensions (shape, color, and number) and they had to figure out the rules behind the sorting mechanism. As no obvious rewards or punishments are involved, the WCST is broadly in line with the traditional construct of cool EF.

Hot EF on the other hand involves goal-directed, future-oriented cognitive processes elicited in contexts that generate emotion, motivation, and a tension between instant gratification and long-term rewards (e.g., Zelazo and Müller, 2002; Zelazo et al., 2005). Hot EF has been posited to include affective cognitive abilities, such as the ability to delay gratification and affective decision making (Zelazo and Müller, 2002; Zelazo and Carlson, 2012). One prominent example is the gambling task (e.g., Iowa Gambling Task; Bechara et al., 2001), which captures important components of hot EF, including reward sensitivity (likeliness of risk modulation when the probabilities of outcomes change) and delay discounting (tendency to choose a smaller, sooner reward over a larger, later reward).

Although current research does not make a convincing case for the distinction of the hot and cool forms, most research prone to agree that EF is relatively more unidimensional in early childhood and specializes into distinct functions with development (Zelazo and Carlson, 2012; Welsh and Peterson, 2014). Basic neural structures involving EF may be the same irrespective of developmental stage; however, important differences exist regarding their maturity. Physiological findings show ongoing nerve fibers' myelination and frontal lobe structures' maturation during adolescence (Klingberg et al., 1999), and speculate that adolescence may constitute a developmentally sensitive period for EF (Giedd, 2004; Blakemore and Choudhury, 2006). Furthermore, different EF components demonstrate various developmental trajectories: age-related improvements seem to occur later as well as more gradually for hot than for cold EF components (Prencipe et al., 2011; Smith et al., 2012). Speculation on the developing brain raises questions about the relationships between the ongoing progression of EF during adolescence and its impact throughout development. Additionally, language experience may cause significant impact to the age related changes in hot and cool EF. There is substantial converging evidence that bilingualism trains EF through its mechanism for language selection (e.g., Bialystok, 2001); and the management of two language systems implicating systematic changes in neural correlates (Bialystok et al., 2005). Moreover, some studies document that bilinguals develop comparatively more rapidly on EF domains such as inhibitory control, working memory, and executive attention than monolinguals (e.g., Hilchey and Klein, 2011; Bialystok, 2017). Nonetheless, most of the studies investigated these connections are either in children or adult populations. Hence, the study of bilingual adolescents may prove particularly valuable.

Aforementioned, empirical evidence suggests a link between EF and important developmental areas such as academic performance and psychological well-being. Indeed, most of these explanations are largely restricted to cool EF. An analysis of six large-scale datasets showed that abilities related to cool EF are an essential indicator of school readiness (Duncan et al., 2007). Other research studied preschoolers (McClelland et al., 2007), and kindergarteners (Blair and Razza, 2007) imply that cool EF along with growth in reading, writing, and mathematics are the major components of academic achievement. In primary school, EF is related to achievement in three subjects including language arts, mathematics, and science (St. Clair-Thompson and Gathercole, 2006). Taken together, research consistently demonstrates a pattern of relationship between children's cool EF and academic outcomes, yet findings in hot EF and especially in adolescence are scarce.

Apart from academic performance, the development of EF and its impact on emotional and behavioral difficulties also draws considerable research attention (e.g., Poon and Ho, 2014). For example, Morgan and Lilienfeld (2000) conducted an extensive meta-analysis on the relationships between cool EF and deviant behavior in adults. The study suggested that antisocial groups perform significantly worse on cool EF involving working memory, planning, and inhibition. For children, some studies have shown that young children with serious problem behaviors exhibit clear deficits in cool EF (e.g., Hughes et al., 2000; Poon and Ho, 2014), suggesting that deficits in cool EF may serve as an early marker of future problematic behaviors (Riggs et al., 2003). Although sparse, some research has examined the relation between hot EF and emotional problems. McIntyre et al. (2006) suggest that high hot EF such as the ability to resist temptation upon school entry predicts teacher-rated prosocial skills and positive relationships in kindergarten.

In sum, reviews emphasize that research on the development of EF has disproportionally focused on the preschool or adult population, leaving out the important developmental period of adolescence. Moreover, although hot and cool EFs can be dissociated in lesioned brains, they typically work together as part of a more general adaptive function. Most studies investigating the development of EF only measure cool component, and research integrating hot and cool EFs would provide a better picture on the interaction and maturation of EF in adolescence, and how they influence two important developmental outcomes: academic performance and psychological adjustment.

### The present study

The present study examined the development of cool and hot EFs in adolescence. It also examined whether (and if so, how) these two aspects of EF are interrelated. Lastly, the relationships between the two facets of EF and academic and psychological adjustment were also examined. There were four hypotheses as follows:

1. Previous studies suggested that age-related improvements occur faster and sooner for cool than hot EF (e.g., Prencipe et al., 2011; Smith et al., 2012). It is hypothesized that age-related improvements will be found in all EF tasks, but improvements on cool EF will occur earlier whereas improvement on hot EF will be more gradual and at slower pace.
2. Neuropsychological evidence on lesioned brains demonstrates a functional dissociation on hot and cool EFs (Zelazo and Carlson, 2012; Welsh and Peterson, 2014). It is hypothesized that hot and cool EFs are separable domains, and measures on hot and cool EFs will not be correlated.
3. According to previous findings on cool EF and academic attainment (Blair and Razza, 2007; Brock et al., 2009; Lan et al., 2011), cool EF is found to be more predictive of academic performance which requires more cognitive processing. The current study expects that cool EF but not hot EF, would account for unique variance in academic performance in particular subject grades, when controlling for general intelligence.
4. Based on previous work on hot EF and emotional difficulties (Kim et al., 2013), it is confirmed that hot EF consistently associates with developmental outcomes that heavily regress on emotional regulation. The current study expects that hot EF but not cool EF, would account for unique variance in emotional problems, when controlling for general intelligence.

## Methodology

### Participants

A total of 136 adolescents were randomly recruited from four secondary schools. Participants ranged from 12 to 17 years old (*M* = 14.45, *SD* = 1.614), with 65 (47.8%) males and 71 (52.2%) females. Participants were divided into six age groups (12.0–12.11, 13.0–13.11, 14.0–14.11, 15.0–15.11, 16.0–16.11, 17.0–17.11 years). Each age group had an approximately equal number of boys and girls. All participants had normal intelligence without suspected brain damage, sensory, psychiatric, or neurological problems, and their first language was Cantonese. In Hong Kong, 95% of its population is ethnically Chinese with Cantonese as the first language while English is the second language that is taught at school. Children with special educational needs were excluded from the study.

### Procedures

The study had two phases: screening and assessment. In the screening phase, participants were administered a standardized subtest used primarily to rule out intellectual disability. During the assessment phase, five EF tasks (four cool EF tasks and one gambling task) were administered individually in a random order. Participants then completed a self-reported questionnaire on demographic background and academic performance. Parents were also invited to complete a questionnaire on participants' psychological well-being.

All assessments were conducted in Cantonese and administered by well-trained research assistants. All parents and adolescents gave informed consent to participate in the study. All participants were informed that their anonymity would be protected and that they could withdraw at any time. A number was issued to each participant for research purposes. Information would be destroyed once the testing was completed. Ethical approval was obtained from the Human Research Ethics Committee at the Education University of Hong Kong.

### Measures

#### General intellectual ability

##### Wechsler Intelligence Scale for Children—Fourth Edition (Hong Kong) (WISC-IV (HK); Wechsler, 2010)

General intellectual ability of the participants was estimated by the oral vocabulary subtest of the WISC-IV(HK) (Wechsler, 2010). The vocabulary subtest has the highest pattern/structure coefficient (*b* = 0.79) to general intelligence among all subtests (Watkins et al., 2006). The participants' vocabulary knowledge was used as a proxy to measure general verbal ability as in previous studies (e.g., McBride-Chang and Suk-Han Ho, 2005; Poon and Ho, 2015). The oral vocabulary subtest included 32 items. Participants were given the target words and required to explain the concepts of these words orally. The test was terminated if the participant scored 0 on five questions in a row. Participants with scaled score 7 or above (mean score = 10, 1 *SD* = 3) were included in the study. The inclusion criteria were to exclude those who might have poor EF merely because of poor intellectual abilities.

#### Measures of cool executive function

##### Backward digit span

Backward digit span subtest from The Test of Specific Learning Difficulties in Reading and Writing for Primary School Students, Second Edition (HKT-P [II]; Chan et al., 2006; Ho et al., 2007) was used to measure working memory. Participants were asked to repeat 2–8 auditorily presented digits in backward order. In order to recall correctly, participants must inhibit current activity (the originally presented digit sequence) and choose relevant information to sustain in working memory.

##### Contingency Naming Test (CNT; Taylor et al., 1990)

The CNT measures attentional control and cognitive flexibility on four rules of increasing difficulty that are applied to a stimulus set of nine practice items and 27 test items. Each stimulus is composed by an outer shape (circle, triangle, or square) of different colors (blue, yellow, or red), and one smaller independent shape (circle, triangle, or square) embedded inside each outer shape. Some of the stimuli include a backwards arrow that appears directly above the outer shape. Participants were asked to name the stimuli according to the rule level under explicitly timed conditions. In level A (attentional control; CNT subtests 1+2), participants were required to name the color (Rule A1) or the shape (Rule A2) of each stimulus. In level B (cognitive flexibility; CNT subtests 3+4), participants were required to switch between naming the stimuli by color or by shape, depending on one attribute (Rule B1) or two attributes (Rule B2).

Unlike other existing measures of EF, the CNT does not demand suppression of automatic responses (Anderson et al., 2001). Instead, participants have to “choose” which of two equally salient responses is appropriate (Kirk et al., 2005). Although the interpretation of CNT scores varies, there is a general consensus that Level A is a test of attention and Level B is a test of cognitive flexibility, which assesses the “ability to consciously switching between responses depending on different attributes” (Dempster and Corkill, 1999, p. 397).

##### Stockings of Cambridge (SOC)

A computerized Stockings of Cambridge (SOC) test was administered to measure goal-setting and planning ability in the visual domain. This test is valid to measure planning ability which is one of the variables of EFs (Hill, 2004; Chiang and Gau, 2014; Palmer et al., 2015). It makes use of three colored balls that can be placed on pegs of three different heights. Participants were required to match the computer's ball pattern, presented on a screen, in a prescribed number of moves, while adhering to a number of specific rules. There are 12 problems, graded in difficulty, with the simplest problems requiring two moves and the most difficult items requiring five moves. Participants were presented two displays, each containing three colored balls arranged differently. The upper display functions as a guideline in which participants have to move the colored balls in the lower display to look exactly the same as the upper one. Three rules are given. First, balls located below another one cannot be moved unless the top ball is removed prior to the action. Second, participants can only move only one ball at a time. Third, the number of allowed moves is limited, and there is no option to reverse moves. Number of correct trials and latency of each trial were recorded.

##### Stroop Color and Word test (Stroop, 1935)

The Stroop Color and Word test is a popular test for measuring inhibition (e.g., Ellis et al., 2009; Dimoska-Di Marco et al., 2011). It involves the presentation of color words in incongruously colored ink (e.g., the word “red” printed in blue ink). Participants are asked to name, as quickly as possible, the ink color of each stimulus word, while attempting to ignore the meaning of the word. This attempt to suppress word meaning in order to name ink color has reliably been shown to result in longer response latencies than those that result from color-naming congruent stimuli (e.g., the word “red” printed in red ink), a phenomenon known as the Stroop effect (MacLeod, 1991). The dependent variable in the Stroop test is either response latency, which is the time in milliseconds between stimulus onset and the participant's response, or an interference score, usually measured as the difference in response latencies between incongruent and congruent stimuli, or between incongruent and colored non-lexical stimuli. Only the interference score was included in the current study to measure participants' inhibitory ability.

#### Measures of hot executive function

A computerized Cambridge Gambling Task (CGT) was administered to capture both reward-related decision making and delay discounting in an authentic setting (Rogers et al., 1999a,b). In the CGT, participants first see 10 boxes colored either in red or blue on the top of the screen. The display of the boxes represents probability of winning. The participant must guess whether a yellow token is hidden in a red or a blue box. In each gambling stage, participants start with a number of points, displayed on the screen, and can select a proportion of these points, displayed in either ascending order (5, 25, 50, 75, up to 95%) or descending order to gamble on their confidence in this judgment. High-/low-pitched sounds prompt regarding a win or loss at the end of every round. When participants finish a trial, a final point total is presented. At the beginning of the first trial, all participants are informed that they are playing for a joint school competition and for those who scored on the top 10 would receive a souvenir as a reward in order to enhance the emotion component of the gambling test. The two outcome measures are risk adjustment and delay aversion. Risk adjustment is the degree to which a subject varies his/her risk-taking in response to the ratio of red to blue boxes on each trial; higher scores represent higher likelihood of response modulation when outcome probability changes. Delay aversion is measured by the difference in percentage bet in ascending verses descending conditions. If participants are unwilling or unable to wait to make a decision, they will be more likely to bet larger amounts when the possible bets are displayed in descending rather than ascending order; higher scores reflect higher impulsiveness.

#### Measures of academic achievement

A self-report questionnaire has been developed in Chinese to obtain background information about participants (e.g., gender, age, grade level) and their parents (e.g., parents' education and family income). Given that there were no culturally valid measures of academic attainment in the Hong Kong context, academic achievement was measured by students' self-report of their numerical grades in three core academic subjects (English, Chinese, and math) as shown on their printed report cards supplied to them during the questionnaire administration. Previous studies of Hong Kong students have also used self-reported grades in these academic subjects as indicators of student achievement (e.g., Chen, 2005, 2008). To minimize possible social desirability bias in participants' responses, participants were assured that their anonymity would be maintained and their participation was voluntary before the distribution of questionnaire.

#### Measures of behavioral and emotional difficulties

##### The Strengths and Difficulties Questionnaire Parent Version (SDQ)

The Strengths and Difficulties Questionnaire (SDQ) Parent Version was used in this study. This is a 25-item measure assessing the potential of having mental health issues. Respondents rate the extent to which item descriptions fit their children well on 3-point Likert scale ranging from 1 (*not true*) to 3 (*certainly true*). Items are summed into five domains: emotional problems (e.g., “Many worries or often seems worried”), conduct problems (e.g., “Generally well behaved, usually does what adults request”), hyperactivity (e.g., “Restless, overactive, cannot stay still for long”), peer problems (e.g., “picked on or bullied by other youth”), and prosocial (e.g., “often offers to help others”) to obtain a composite score from 25 to 75. The parent-version SDQ was pre-distributed along with consent aimed to provide a more aggregated and reliable view of the above domains. The domain score is directly proportional to the probability that the participant suffers from psychological abnormalities. Reliability and validity of the SDQ are generally satisfactory (Goodman, 2001; Stone et al., 2010).

### Statistical analyses

Initial analyses were performed to examine the demographic characteristics of the sample. One-way analysis of covariance (ANCOVA) with general intellectual ability as covariate were computed for main effect of Age on EF components. Bonferroni correction was also employed for group comparison within each cool and hot EF components with critical value set at *p* < 0.05. Pearson's correlation analysis controlling age, general intellectual ability, and socioeconomic status (SES) variables including family income and father's and mother's education level was conducted to examine correlations among and between cool and hot EF variables. In projecting major developmental outcomes, two hierarchical Regression analyses were computed to examine EF-components in predicting academic performance and psychological adjustment.

## Results

### Descriptive statistics

Data were analyzed with the SPSS version 21 for Windows, and were inspected for normality to ensure that the assumptions of parametric statistics were met before analyses were performed. Tables 1, 2 summarize the descriptive statistics for gender, age, general intellectual ability, educational level, average grades, and grades for specific subjects (Chinese, English, and Mathematics), parents' educational level, and family income. Group differences were found in education level [*F*_(5, 179)_ = 140.29, *p* < 0.001, η_*p*_^2^ = 0.90], Chinese grades [*F*_(5, 137)_ = 2.64, *p* < 0.05, $n_{p}^{2}$ = 0.15], father's education level [*F*_(5, 137)_ = 2.89, *p* < 0.05, η_*p*_^2^ = 0.16], and mother's education level [*F*_(5, 137)_ = 9.42, *p* = 0.00, η_*p*_^2^ = 0.39]. No significant difference was found in general intellectual ability [*F*_(5, 137)_ = 1.56, *p* = 0.18, η_*p*_^2^ = 0.05], average grade [*F*_(5, 137)_ = 0.46, *p* = 0.81, η_*p*_^2^ = 0.03], English grades [*F*_(5, 137)_ = 1.10, *p* = 0.37, η_*p*_^2^ = 0.03], Mathematics grades [*F*_(5, 137)_ = 0.58, *p* = 0.72, η_*p*_^2^ = 0.04], or family income [*F*_(5, 124)_ = 0.38, *p* = 0.86, η_*p*_^2^ = 0.03). Preliminary analyses revealed no significant difference in gender [$\chi_{\left(5,\;\text{N}=185\right)}^{2}$ = 1.96, *p* = 0.86] or parents' marital status [$\chi_{\left(15,\;\text{N}=142\right)}^{2}$ = 22.12, *p* = 0.11; see Table 3 for parents' marital status]. Thus, data were combined across gender for purposes of subsequent analyses, and participants' general intellectual ability scores were entered as a covariate as EF is a cognitive factor relating to intellectual ability.

**Table 1:** Characteristics of adolescent according to age with IQ as covariance.

	**Age**						***F***	***P***	**$n_{p}^{2}$**	***Post-hoc***
	**12**	**13**	**14**	**15**	**16**	**17**				
	**(*N* = 27)**	**(*N* = 27)**	**(*N* = 35)**	**(*N* = 31)**	**(*N* = 39)**	**(*N* = 26)**				
General intellectual ability^a^^,^^b^	9.96	10.38	10.52	10.78	11.23	11.86	1.56	0.18	0.05	
	(2.30)	(3.49)	(2.64)	(2.45)	(2.64)	(1.77)				
Education level^c^	7.08	6.57	8.14	8.23	9.48	11.00	140.29	0.00^***^	0.90	17>16>15>14>13>12
	(0.28)	(0.54)	(0.54)	(0.60)	(0.60)	(0.00)				
Average grades^d^	2.46	3.00	2.93	2.85	3.05	2.86	0.46	0.81	0.03	
	(0.88)	(0.82)	(1.49)	(1.28)	(1.12)	(0.86)				
Chinese grades^d^	2.08	2.86	2.64	2.69	3.19	2.50	2.64	0.03^*^	0.15	16>12
	(0.95)	(0.69)	(1.08)	(1.03)	(0.93)	(0.76)				
English grades^d^	2.85	3.14	3.00	3.23	3.14	2.43	1.10	0.37	0.07	
	(1.14)	(1.22)	(0.68)	(1.09)	(1.15)	(0.85)				
Math grades^d^	2.38	3.00	2.86	2.85	3.05	2.86	0.58	0.72	0.04	
	(0.96)	(0.82)	(1.41)	(1.28)	(1.12)	(.86)				
Edu level father^e^	2.00	1.71	2.36	2.00	1.62	1.79	2.89	0.02^*^	0.16	16>14
	(0.58)	(0.49)	(0.63)	(0.82)	(0.50)	(0.43)				
Edu level mother^e^	2.38	2.14	2.21	2.08	1.71	1.29	9.42	0.00^***^	0.39	17>15,14,13,12; 16>14,12
	(0.51)	(0.38)	(0.43)	(0.49)	(0.46)	(0.47)				
Family income	4.46	4.71	5.79	5.08	5.29	6.36	0.38	0.86	0.03	
	(3.38)	(1.80)	(5.162)	(2.33)	(3.35)	(4.60)				

a *Wechsler Intelligence Scale for Children**—**Fourth Edition*,

b *Scaled score*,

c *American School System*,

d *Percentage Grade System*,

e *1, First to sixth grades; 2, Seventh to twelfth grades; 3, Associate/Bachelor degree; 4, Postgraduate*.

* *p < 0.05*,

*** *p < 0.001*.

**Table 2:** Cross-tabulation of gender and age groups.

**Gender**	**Age**							**χ^2^**	**Φ**
	**12**	**13**	**14**	**15**	**16**	**17**	**Total**		
	**(*N* = 27)**	**(*N* = 27)**	**(*N* = 35)**	**(*N* = 31)**	**(*N* = 39)**	**(*N* = 26)**	**(*N* = 26)**		
Male	14	15	20	15	18	11	93	1.96	0.10
	(13.57)	(13.57)	(17.59)	(15.58)	(19.60)	(13.07)		*p* = 0.86	
Female	13	12	15	16	21	15	92		
	(13.43)	(13.43)	(13.41)	(15.42)	(19.39)	(12.93)			
Total	27	27	35	31	39	26	185		

*Total valid case: N = 185, Numbers in brackets represent Expected Count*.

**Table 3:** Cross-tabulation of parents marital status and age groups.

**Parents marital status**	**Age**							**χ^2^**	**Φ**
	**12**	**13**	**14**	**15**	**16**	**17**	**Total**		
	**(*N* = 24)**	**(*N* = 21)**	**(*N* = 23)**	**(*N* = 23)**	**(*N* = 30)**	**(*N* = 21)**			
Widowed	3	0	0	0	0	2	5	22.12	0.40
	(0.85)	(0.74)	(0.81)	(0.81)	(1.06)	(0.74)		*p* = 0.11	
Separated	0	0	0	1	1	1	3		
	(0.51)	(0.44)	(0.49)	(0.49)	(0.63)	(0.44)			
Divorced	5	5	2	1	8	2	23		
	(3.89)	(3.40)	(3.73)	(3.73)	(4.86)	(3.40)			
Married	16	16	21	21	21	16	111		
	(18.76)	(16.42)	(17.98)	(17.98)	(23.45)	(16.42)			
Total	24	21	23	23	30	21	142		

*Total valid case: N = 142 (M = 93; F = 92), Numbers in brackets represent Expected Count*.

#### Relations among executive function measures

The construct validity of hot and cool EFs was examined by partial Pearson's correlation analysis controlling age, general intellectual ability, socioeconomic status (SES) related variables including family incomes, father's and mother's educational levels (see Table 4). These correlations demonstrate the divergent relationships among hot and cool EF measures. Results revealed positive internal correlations among cool EF measures. Significant positive correlation was found between Backward Digit Span and CNT attentional control (*r* = 0.180, *p* < 0.05), followed by a strong correlation trend between Stroop and CNT attentional control (*r* = 0.166, *p* = 0.07) within cool EF domains. None of the hot EF measures were significantly or marginally inter-correlated or correlated.

**Table 4:** Correlations among cool and hot executive function variables after controlling age, IQ, family income, and family education level.

	**Cool EF**					**Hot EF**	
	**Backward digit-span**	**CNT-attention control**	**CNT-cognitive flexibility**	**Stroop-interference**	**SOC-problems solved inminimum moves**	**CGT-risk adjustment**	**CGT-Delayaversion**
Backward							
digit-span							
CNT-	0.18^*^						
attention control							
CNT-	−0.00	0.14					
cognitive flexibility							
Stroop-	−0.01	0.17^a^	−0.00				
interference							
SOC-	0.05	0.05	0.07	0.05			
problems solved in minimum moves							
CGT-	−0.06	−0.07	0.05	0.04	−0.03		
risk adjustment							
CGT-delay	−0.03	−0.01	−0.07	−0.08	0.05	−0.07	
aversion							

*CNT, Contingency Naming Test; CGT, Cambridge Gambling Task; SOC, Stockings of Cambridge*.

* *p < 0.05*.

a *p = 0.07*.

#### Cool executive function

In order to investigate the developmental trajectory of both hot and cool EFs, a one-way analysis of covariance (ANCOVA) was adopted to compare group differences among the six age groups (12.0–12.11, 13.0–13.11, 14.0–14.11, 15.0–15.11, 16.0–16.11, and 17.0–17.11 years) on each EF measure. Participants' general intellectual ability scores were entered as a covariate.

##### Backward Digit Span

**Figure 1:**
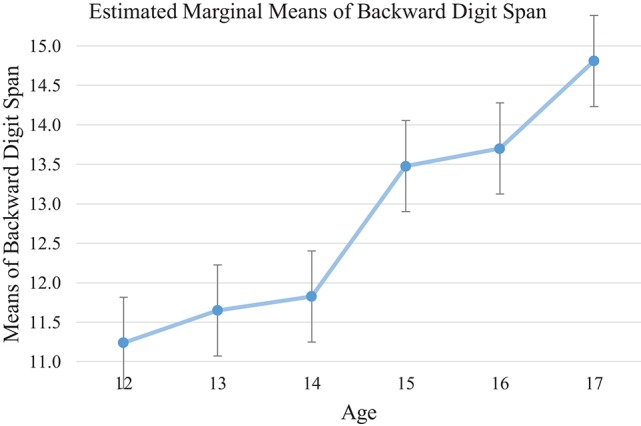
Mean of each age group on backward digit span test. Processing Speed improves across different ages with a spurt between age of 14 and 15.

Backward Digit Span was used to measure working memory. Significant age effects was identified [*F*_(5, 135)_ = 6.26, *p* < 0.001, η_*p*_^2^ = 0.19] with older adolescents demonstrating higher working memory capacity. Bonferroni *post-hoc* analysis showed that 17-year-olds showed significant higher capacity than the 12- to 14-year-old groups (12-year-old: mean differences = 3.63, 95% CI [1.22, 6.05], *p* < 0.01; 13-year-old: mean differences = 3.22, 95% CI [0.68, 5.75], *p* < 0.01; 14-year-old: mean differences = 3.63, 95% CI [0.60, 5.46] *p* < 0.01). Sixteen year-olds performed better than the12-year-olds (mean differences = 2.50, 95% CI [0.32, 4.69], *p* < 0.05), yet 15 year-olds showed a trend of outperforming the 12-year-olds (mean differences = 2.27, 95% CI [−0.050, 4.58], *p* = 0.06). These results might suggested a developmental spurt in working memory capacity at around age 15 (see Tables 5, 6, and Figure 1).

**Table 5:** Means, standard deviations according to cool and hot executive function tasks for age groups with IQ as covariance.

	**Age**						**ANOVA**	
	**12**	**13**	**14**	**15**	**16**	**17**	***F***	***Post-hoc***
	**(*N* = 27)**	**(*N* = 27)**	**(*N* = 35)**	**(*N* = 31)**	**(*N* = 39)**	**(*N* = 26)**		**(*N* = 26)**
**COOL EF**								
Backward	11.24	11.65	11.83	13.48	13.70	14.81	6.26^***^	17>14,13,12;
digit-span	(2.52)	(2.68)	(2.77)	(2.52)	(2.68)	(2.79)		16,15>12
CNT-	11.32	11.90	11.63	12.56	13.35	14.56	5.79^***^	17>15,14,13,12;
attention control	(1.81)	(2.22)	(2.17)	(2.56)	(2.64)	(2.94)		16>12
CNT-	3.52	3.73	3.77	4.07	4.66	4.73	4.03^**^	17,16>12
cognitive flexibility	(1.05)	(1.00)	(1.36)	(.91)	(1.34)	(1.09)		
Stroop-	11.44	11.30	11.83	12.70	13.64	13.29	3.00^*^	16>13,12
interference	(3.93)	(1.42)	(1.83)	(1.40)	(2.60)	(3.32)		
SOC-	7.48	8.10	7.87	8.17	8.53	10.05	3.45^**^	17>15,14,13,12
problem solved in minimum move	(2.10)	(2.19)	(1.96)	(2.27)	(2.22)	(1.80)		
**HOT EF**								
CGT-	0.69	0.95	1.44	1.19	0.88	0.54	3.87^**^	14>12; 14>17
risk adjustment	(0.66)	(0.75)	(0.82)	(0.94)	(0.92)	(0.53)		
CGT-	0.18	0.22	0.31	0.33	0.22	0.21	1.30	
delay aversion	(0.26)	(0.37)	(0.20)	(0.29)	(0.25)	(0.19)		

*CNT, Contingency Naming Test; CGT, Cambridge Gambling Task; SOC, Stockings of Cambridge*.

* *p < 0.05*,

** *p < 0.01*,

*** *p < 0.001*.

**Table 6:** Result of Bonferroni correction of backward digit-span according to age groups.

**Age (i)**	**Age (j)**	**Mean difference**	**Std. error**	**Sig.**	**95% confidence interval for difference**	
		**(i–j)**			**Lower bound**	**Lower bound**
12	13	−0.42	0.80	1.00	−2.81	1.98
	14	−0.61	0.77	1.00	−2.91	1.70
	15	−2.27	0.77	0.06	−4.58	0.05
	16	−2.50^*^	0.73	0.01	−4.69	−0.32
	17	−3.63^***^	0.81	0.00	−6.05	−1.22
13	12	0.42	0.80	1.00	−1.98	2.81
	14	−0.19	0.82	1.00	−2.63	2.25
	15	−1.85	0.82	0.38	−4.30	0.59
	16	−2.09	0.78	0.12	−4.41	0.23
	17	−3.22^**^	0.85	0.00	−5.75	−0.68
14	12	0.61	0.77	1.00	−1.70	2.91
	13	0.19	0.82	1.00	−2.25	2.63
	15	−1.66	0.79	0.55	−4.01	0.69
	16	−1.90	0.74	0.18	−4.12	0.32
	17	−3.03^**^	0.81	0.00	−5.46	−0.60
15	12	2.27	0.77	0.06	−0.05	4.58
	13	1.85	0.82	0.38	−0.59	4.30
	14	1.66	0.79	0.55	−0.69	4.01
	16	−0.24	0.74	1.00	−2.45	1.98
	17	−1.37	0.81	1.00	−3.79	1.06
16	12	2.50^*^	0.73	0.01	0.32	4.69
	13	2.09	0.78	0.12	−0.23	4.41
	14	1.90	0.74	0.18	−0.32	4.12
	15	0.24	0.74	1.00	−1.98	2.45
	17	−1.13	0.76	1.00	−3.41	1.14
17	12	3.63^***^	0.81	0.00	1.22	6.05
	13	3.22^**^	0.85	0.00	0.68	5.75
	14	3.03^**^	0.81	0.00	0.60	5.46
	15	1.36	0.81	1.00	−1.06	3.79
	16	1.13	0.76	1.00	−1.14	3.41

* *p < 0.05*,

** *p < 0.01*,

*** *p < 0.001*.

##### Contingency Naming Test attentional control

**Figure 2:**
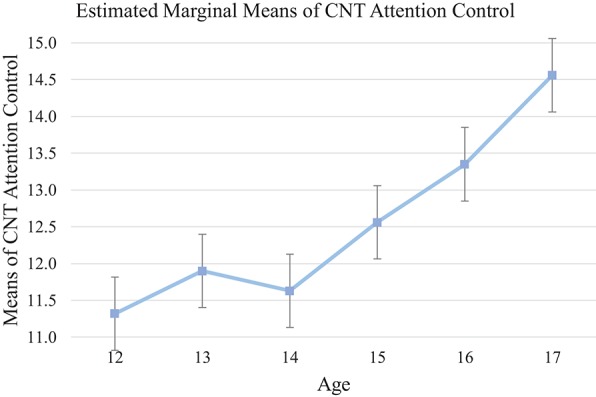
Mean of each age group on CNT (attentional control: subtest 1+2). Attention control improved from age 14 suddenly with steady pattern.

Analysis of the basic processing component (subtests 1+2) of the CNT attentional control demonstrated a significant difference across the age range, [*F*_(5, 134)_ = 5.79, *p* < 0.001, η_*p*_^2^ = 0.18]. *Post-hoc* comparisons revealed significantly better results for the 17-year-olds compared with younger participants (15-year-old: mean difference = 2.06, 95% CI [−0.14, 4.26], *p* = 0.08; 14-year-old: mean differences = 3.01, 95% CI [0.80, 5.21]; 13-year-old: mean differences = 2.76, 95% CI [0.46, 5.06]; 12-year-old: mean differences = 3.34, 95% CI [1.15, 5.53], *p* < 0.01). Moreover, the 16-year-olds scored significant higher than 12-year-olds (mean differences = 2.11, 95% CI [0.11, 4.41], *p* = 0.03). Profile plot revealed a steep upward slope from age 14 onwards with significant improvement topped at age 17 suggesting sudden developmental growth in attentional control between 14 and 17 years old (see Tables 5, 7, and Figure 2).

**Table 7:** Result of Bonferroni correction of CNT attentional control according to age groups.

**Age (i)**	**Age (j)**	**Mean difference**	**Std. error**	**Sig.**	**95% Confidence interval for difference**	
		**(i–j)**			**Lower bound**	**Lower bound**
12	13	−0.59	0.73	1.00	−2.76	1.58
	14	−0.34	0.70	1.00	−2.43	1.76
	15	−1.29	0.70	1.00	−3.38	0.81
	16	−2.11^*^	0.67	0.03	−4.11	−0.11
	17	−3.34^***^	0.73	0.00	−5.53	−1.15
13	12	0.59	0.73	1.00	−1.58	2.76
	14	0.25	0.74	1.00	−1.97	2.46
	15	−0.70	0.74	1.00	−2.92	1.52
	16	−1.53	0.71	0.50	−3.65	0.60
	17	−2.76^**^	0.77	0.01	−5.06	−0.46
14	12	0.34	0.70	1.00	−1.76	2.43
	13	−0.25	0.74	1.00	−2.46	1.97
	15	−0.95	0.71	1.00	−3.08	1.19
	16	−1.77	0.68	0.15	−3.80	0.26
	17	−3.01^***^	0.74	0.00	−5.21	−0.80
15	12	1.29	0.70	1.00	−0.81	3.38
	13	0.70	0.74	1.00	−1.52	2.92
	14	0.95	0.71	1.00	−1.19	3.08
	16	−0.83	0.68	1.00	−2.85	1.20
	17	−2.06	0.74	0.09	−4.26	0.14
16	12	2.11^*^	0.67	0.03	0.11	4.11
	13	1.53	0.71	0.50	−0.60	3.65
	14	1.77	0.68	0.15	−0.26	3.80
	15	0.83	0.68	1.00	−1.20	2.85
	17	−1.23	0.70	1.00	−3.31	0.84
17	12	3.34^***^	0.73	0.00	1.15	5.53
	13	2.76^**^	0.77	0.01	0.46	5.06
	14	3.01^***^	0.74	0.00	0.80	5.21
	15	2.06	0.74	0.09	−0.14	4.26
	16	1.23	0.70	1.00	−0.84	3.31

* *p < 0.05*,

** *p < 0.01*,

*** *p < 0.001*.

##### Contingency Naming Test cognitive flexibility

**Figure 3:**
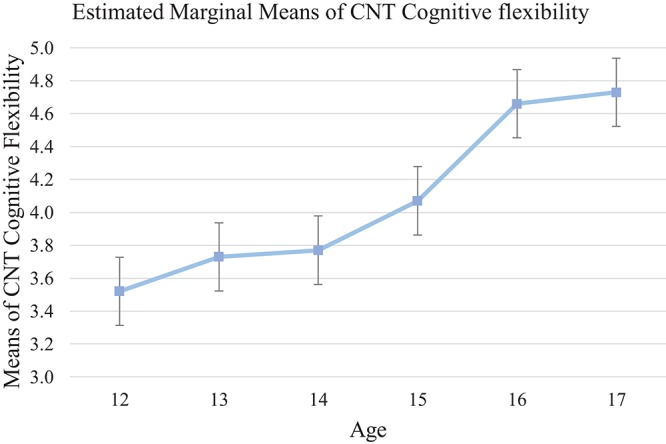
Mean of each age group on CNT (cognitive flexibility: subtest 3+4). Cognitive flexibility shows steady growth throughout different ages.

Results from CNT cognitive flexibility (subtests 3+4) showed similar pattern as CNT attentional control. Significant age effects were identified [*F*_(5, 133)_ = 4.03, *p* = 0.001, η_*p*_^2^ = 0.13] with older adolescents demonstrated better flexibility. *Post-hoc* analysis showed that participants at age 16 and older performed significantly better than the 12-year-olds (17-year-old: mean differences = 1.16, 95% CI [0.12, 2.20], *p* < 0.05; 16-year-old: mean differences = 1.10, 95% CI [0.142, 2.06], *p* < 0.05). This pattern indicated that a steady growth in cognitive flexibility was observed from age 12 to 17 (see Tables 5, 8, and Figure 3).

**Table 8:** Result of Bonferroni correction of CNT cognitive flexibility according to age groups.

**Age (i)**	**Age (j)**	**Mean difference**	**Std. error**	**Sig.**	**95% Confidence interval for difference**	
		**(i–j)**			**Lower bound**	**Lower bound**
12	13	−0.20	0.35	1.00	−1.24	0.83
	14	−0.24	0.33	1.00	−1.23	0.76
	15	−0.53	0.33	1.00	−1.53	0.47
	16	−1.10^*^	0.32	0.01	−2.06	−0.14
	17	−1.16^*^	0.35	0.02	−2.20	−0.12
13	12	0.20	0.35	1.00	−0.83	1.24
	14	−0.03	0.35	1.00	−1.08	1.02
	15	−0.33	0.35	1.00	−1.38	0.73
	16	−0.90	0.34	0.14	−1.91	0.12
	17	−0.95	0.37	0.16	−2.04	0.14
14	12	0.24	0.33	1.00	−0.76	1.23
	13	0.03	0.35	1.00	−1.02	1.08
	15	−0.29	0.34	1.00	−1.31	0.72
	16	−0.86	0.33	0.13	−1.84	0.11
	17	−0.92	0.35	0.15	−1.97	0.13
15	12	0.53	0.33	1.00	−0.47	1.53
	13	0.33	0.35	1.00	−0.73	1.38
	14	0.29	0.34	1.00	−0.72	1.31
	16	−0.57	0.32	1.00	−1.54	0.40
	17	−0.63	0.35	1.00	−1.67	0.42
16	12	1.10^*^	0.32	0.01	0.14	2.06
	13	0.90	0.34	0.14	−0.12	1.91
	14	0.86	0.33	0.13	−0.11	1.84
	15	0.57	0.32	1.00	−0.40	1.54
	17	−0.06	0.33	1.00	−1.05	0.94
17	12	1.16^*^	0.35	0.02	0.12	2.20
	13	0.95	0.37	0.16	−0.14	2.04
	14	0.92	0.35	0.15	−0.13	1.97
	15	0.63	0.35	1.00	−0.42	1.67
	16	0.06	0.33	1.00	−0.94	1.05

* *p < 0.05*.

##### Stockings of Cambridge

**Figure 4:**
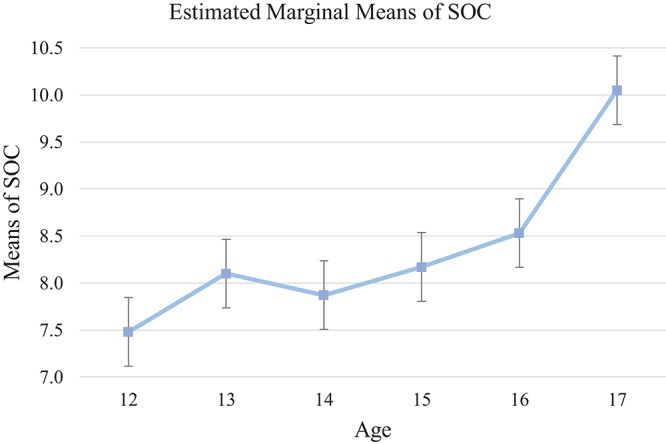
Mean of each age group on SOC Test. SOC shows flat development from age 12 to age 16 and developmental spurt at age 17.

The SOC measures planning ability in the visual domain. A significant effect of age was identified [*F*_(5, 136)_ = 3.45, *p* < 0.01, η_*p*_^2^ = 0.11]. Specifically, the 17-year-old group showed significantly better planning ability. *Post-hoc* analysis showed that the 17-year-old group performed significantly better than 12- (mean differences = 2.46, 95% CI [0.55, 4.36], *p* < 0.01) and 14-year-olds (mean differences = 2.10, 95% CI [0.18, 4.02], *p* < 0.05) with marginal trend of improvement compared with 13- (mean differences = 1.87, 95% CI [−0.10, 3.83], *p* = 0.08) and 15-year-old (mean differences = 1.81, 95% CI [−0.11, 3.73], *p* = 0.08). However, there was no significant group difference between the 12- to 16-year-old groups (*p*s > 0.05). This pattern indicated flat development from age 12 to 16 and a developmental spurt at age 17 (see Tables 5, 9, and Figure 4).

**Table 9:** Result of Bonferroni correction of SOC problem solved in minimum moves according to age groups.

**Age (i)**	**Age (j)**	**Mean difference**	**Std. error**	**Sig.**	**95% confidence interval for difference**	
		**(i–j)**			**Lower bound**	**Lower bound**
12	13	−0.59	0.63	1.00	−2.46	1.28
	14	−0.36	0.61	1.00	−2.18	1.47
	15	−0.65	0.61	1.00	−2.47	1.18
	16	−0.98	0.58	1.00	−2.71	0.75
	17	−2.46^**^	0.64	0.00	−4.36	−0.55
13	12	0.59	0.63	1.00	−1.28	2.46
	14	0.23	0.64	1.00	−1.67	2.14
	15	−0.06	0.64	1.00	−1.96	1.85
	16	−0.39	0.60	1.00	−2.19	1.41
	17	−1.87	0.66	0.08	−3.83	0.10
14	12	0.36	0.61	1.00	−1.47	2.18
	13	−0.23	0.64	1.00	−2.14	1.67
	15	−0.29	0.62	1.00	−2.15	1.57
	16	−0.62	0.59	1.00	−2.38	1.13
	17	−2.10^*^	0.64	0.02	−4.02	−0.18
15	12	0.65	0.61	1.00	−1.18	2.47
	13	0.06	0.64	1.00	−1.85	1.96
	14	0.29	0.62	1.00	−1.57	2.15
	16	−0.33	0.59	1.00	−2.08	1.42
	17	−1.81	0.64	0.08	−3.73	0.11
16	12	0.98	0.58	1.00	−0.75	2.71
	13	0.39	0.60	1.00	−1.41	2.19
	14	0.62	0.59	1.00	−1.13	2.38
	15	0.33	0.59	1.00	−1.42	2.08
	17	−1.48	0.60	0.23	−3.28	0.32
17	12	2.46^**^	0.64	0.00	0.55	4.36
	13	1.87	0.66	0.08	−0.10	3.83
	14	2.10^*^	0.64	0.02	0.18	4.02
	15	1.81	0.64	0.08	−0.11	3.73
	16	1.48	0.60	0.23	−0.32	3.28

* *p < 0.05*,

** *p < 0.01*.

##### Stroop test

**Figure 5:**
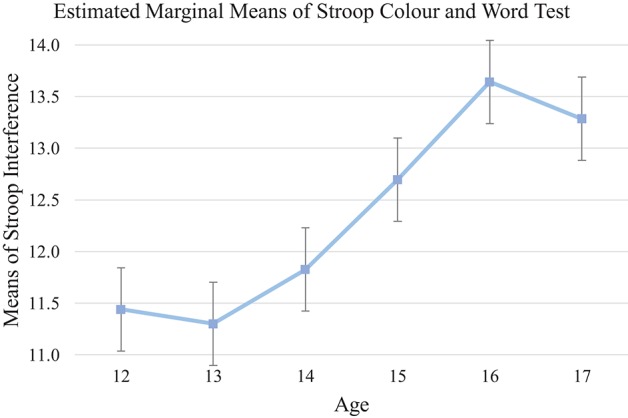
Mean of each age group on Stroop Color and Word Test. Interference control (response inhibition) improved from age 13 to age 16 but shows a downward trend at age 17.

The interference score was used to measure the ability to inhibit dominant or automatic responses. Analysis of Stroop interference demonstrated a significant growth of this ability across the age range, [*F*_(5, 133)_ = 3.00, *p* < 0.05, η_*p*_^2^ = 0.10], with older adolescents demonstrating better inhibition ability. *Post-hoc* analysis showed a steady development from 13-year-olds to 16-year-olds. No significant differences within all age groups (*ps* > 0.05) were observed. However, 16-year-old group preformed marginally better than 12 (mean differences = 2.15, 95% CI [−0.05, 4.35], *p* = 0.06) and 13-year-olds (mean differences = 2.30, 95% CI [−0.04, 4.63], *p* = 0.060). This pattern suggested a steady growth of response inhibition from age 13 to 16 (see Table 5, 10, and Figure 5).

**Table 10:** Result of Bonferroni correction of stroop-interference performance according to age groups.

**Age (i)**	**Age (j)**	**Mean difference**	**Std. error**	**Sig.**	**95% confidence interval for difference**	
		**(i–j)**			**Lower bound**	**Upper bound**
12	13	0.15	0.79	1.00	−2.22	2.51
	14	−0.36	0.76	1.00	−2.65	1.92
	15	−1.22	0.77	1.00	−3.51	1.07
	16	−2.15	0.74	0.06	−4.35	0.05
	17	−1.77	0.80	0.42	−4.16	0.62
13	12	−0.15	0.79	1.00	−2.51	2.22
	14	−0.51	0.81	1.00	−2.92	1.90
	15	−1.37	0.81	1.00	−3.79	1.05
	16	−2.29	0.78	0.06	−4.63	0.04
	17	−1.92	0.84	0.36	−4.43	0.59
14	12	0.36	0.76	1.00	−1.92	2.65
	13	0.51	0.81	1.00	−1.90	2.92
	15	−0.86	0.78	1.00	−3.19	1.47
	16	−1.78	0.75	0.27	−4.02	0.45
	17	−1.41	0.81	1.00	−3.82	1.00
15	12	1.22	0.77	1.00	−1.07	3.51
	13	1.37	0.81	1.00	−1.05	3.79
	14	0.86	0.78	1.00	−1.47	3.19
	16	−0.93	0.74	1.00	−3.15	1.30
	17	−0.55	0.80	1.00	−2.95	1.85
16	12	2.15	0.74	0.06	−0.05	4.35
	13	2.29	0.78	0.06	−0.04	4.63
	14	1.78	0.75	0.27	−0.45	4.02
	15	0.93	0.74	1.00	−1.30	3.15
	17	0.38	0.76	1.00	−1.90	2.66
17	12	1.77	0.80	0.42	−0.62	4.16
	13	1.92	0.84	0.36	−0.59	4.43
	14	1.41	0.81	1.00	−1.00	3.82
	15	0.55	0.80	1.00	−1.85	2.95
	16	−0.38	0.76	1.00	−2.66	1.90

#### Hot executive function

##### Cambridge Gambling Task risk adjustment

**Figure 6:**
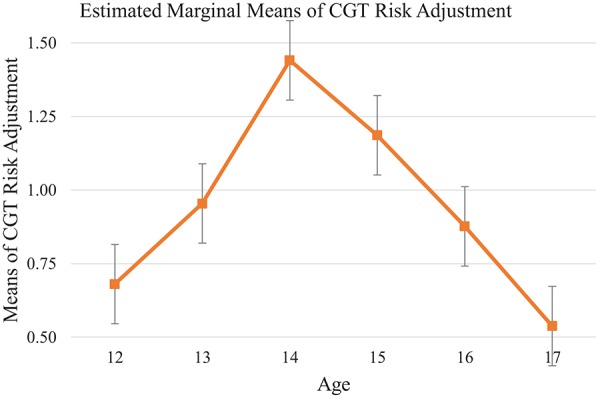
Mean of each age group on CGT Test: risk adjustment. Risk adjustment shows bell shape pattern reaching the top at age 14.

CGT risk adjustment measures the degree to which a participant varies his/her risk-taking in response to the ratio of red to blue boxes on each trial. Different from the steady growth observed in cool EF tests, analysis of CGT risk adjustment showed a bell shape (see Table 11, Figure 6) across the age groups, [*F*_(5, 135)_ = 3.87, *p* < 0.01, η_*p*_^2^ = 0.13]. Profile plot showed a significant upward slope from age 12 and a peak at age 14 (mean differences = 0.76, 95% CI [0.06, 1.46], *p* < 0.05). A downward slope started from age 14 to 17, suggesting a significant decline in performance between ages 14 and 17 (mean differences = −0.91, 95% CI [−1.64, −0.18], *p* < 0.01). This pattern indicated that the 14-year-old group exhibited the highest risk taking tendency, and this propensity declined after age 14.

**Table 11:** Result of Bonferroni correction of CGT risk adjustment according to age groups.

**Age (i)**	**Age (j)**	**Mean difference**	**Std. error**	**Sig.**	**95% confidence interval for difference**	
		**(i–j)**			**Lower bound**	**Lower bound**
12	13	−0.27	0.24	1.00	−0.99	0.44
	14	−0.76	0.23	0.02	−1.46	−0.06
	15	−0.50	0.23	0.51	−1.20	0.20
	16	−0.19	0.22	1.00	−0.85	0.47
	17	0.15	0.24	1.00	−0.58	0.88
13	12	0.27	0.24	1.00	−0.44	0.99
	14	−0.49	0.24	0.68	−1.21	0.23
	15	−0.23	0.24	1.00	−0.95	0.49
	16	0.08	0.23	1.00	−0.60	0.76
	17	0.42	0.25	1.00	−0.32	1.17
14	12	0.76^*^	0.23	0.02	0.06	1.46
	13	0.49	0.24	0.68	−0.23	1.21
	15	0.26	0.24	1.00	−0.45	0.96
	16	0.57	0.22	0.17	−0.10	1.23
	17	0.91^**^	0.24	0.00	0.18	1.64
15	12	0.50	0.23	0.51	−0.20	1.20
	13	0.23	0.24	1.00	−0.49	0.95
	14	−0.26	0.24	1.00	−0.96	0.45
	16	0.31	0.22	1.00	−0.35	0.97
	17	0.65	0.24	0.12	−0.07	1.38
16	12	0.19	0.22	1.00	−0.47	0.85
	13	−0.08	0.23	1.00	−0.76	0.60
	14	−0.57	0.22	0.17	−1.23	0.10
	15	−0.31	0.22	1.00	−0.97	0.35
	17	0.34	0.23	1.00	−0.34	1.02
17	12	−0.15	0.24	1.00	−0.88	0.58
	13	−0.42	0.25	1.00	−1.17	0.32
	14	−0.91	0.24	0.00	−1.64	−0.18
	15	−0.65	0.24	0.12	−1.38	0.07
	16	−0.34	0.23	1.00	−1.02	0.34

* *p < 0.05*,

** *p < 0.01*.

##### Cambridge Gambling Task delay aversion

**Figure 7:**
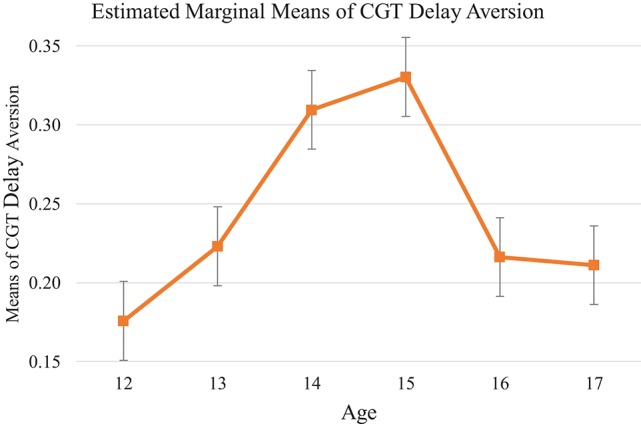
Mean of each age group on CGT Test: Delay Aversion. Choice impulsivity shows bell shape pattern reaching the top at age 15. Significant developmental spurt and drops were found before age 14 and after age 15.

CGT delay aversion measures the difference between the risk-taking score in descending and ascending conditions. The higher the score, the greater preference for smaller-immediate over larger-delayed rewards (choice impulsivity). Similar with CGT risk adjustment, there was a bell shape across the age groups, despite no significant age difference was identified [*F*_(5, 135)_ = 1.30, *p* = 0.27, η_*p*_^2^ = 0.05; see Table 12, Figure 7]. Data plotted from *post-hoc* analysis found that there was an ascending trend from the 12-year-old group and a peak at age 15 (*ps* > 0.05). A descending pattern started from age 15 to 17, although the downward slope was not significant (*ps* > 0.05). This pattern might imply a weaker preference for smaller-immediate over larger-delayed rewards after age 15.

**Table 12:** Result of Bonferroni correction of CGT delay aversion moves according to age groups.

**Age (i)**	**Age (j)**	**Mean difference**	**Std. error**	**Sig.**	**95% confidence interval for difference**	
		**(i–j)**			**Lower bound**	**Lower bound**
12	13	−0.05	0.08	1.00	−0.29	0.18
	14	−0.14	0.08	0.98	−0.37	0.09
	15	−0.17	0.08	0.47	−0.40	0.06
	16	−0.06	0.07	1.00	−0.28	0.16
	17	−0.07	0.08	1.00	−0.31	0.17
13	12	0.05	0.08	1.00	−0.18	0.29
	14	−0.09	0.08	1.00	−0.33	0.15
	15	−0.11	0.08	1.00	−0.35	0.12
	16	−0.01	0.08	1.00	−0.23	0.22
	17	−0.01	0.08	1.00	−0.26	0.23
14	12	0.14	0.08	0.98	−0.09	0.37
	13	0.09	0.08	1.00	−0.15	0.33
	15	−0.03	0.08	1.00	−0.26	0.21
	16	0.08	0.07	1.00	−0.14	0.30
	17	0.08	0.08	1.00	−0.17	0.32
15	12	0.17	0.08	0.47	−0.06	0.40
	13	0.11	0.08	1.00	−0.12	0.35
	14	0.03	0.08	1.00	−0.21	0.26
	16	0.11	0.07	1.00	−0.11	0.33
	17	0.10	0.08	1.00	−0.14	0.34
16	12	0.06	0.07	1.00	−0.16	0.28
	13	0.01	0.08	1.00	−0.22	0.23
	14	−0.08	0.07	1.00	−0.30	0.14
	15	−0.11	0.07	1.00	−0.33	0.11
	17	−0.01	0.08	1.00	−0.23	0.22
17	12	0.07	0.08	1.00	−0.17	0.31
	13	0.01	0.08	1.00	−0.23	0.26
	14	−0.08	0.08	1.00	−0.32	0.17
	15	−0.10	0.08	1.00	−0.34	0.14
	16	0.01	0.08	1.00	−0.22	0.23

### Predicting major developmental outcomes

#### Academic performance

A hierarchical regression analysis controlling for general intellectual ability was conducted to examine which cool or hot EF measures best predict the two developmental outcomes: (1) academic performance and (2) psychological adjustment. Table 13 showed that Stroop (*B* = 0.26, *t* = 3.21, *p* < 0.05, *R*^2^ = 0.15) and CNT cognitive flexibility (*B* = −0.246, *t* = −2.995, *p* < 0.05, *R*^2^ = 0.15) accounted for an additional 15.2% of the variance in predicting average grade. In predicting Mathematics grade, Stroop (*B* = 0.231, *t* = 2.799, *p* < 0.05, *R*^2^ = 0.10) and CNT cognitive flexibility (*B* = −0.254, *t* = −3.084, *p* < 0.05, *R*^2^ = 0.10) were also significant predictors after controlling for general intellectual ability, and accounted for an additional variance of 10% of Maths grade (see Table 14). No hot EF variables associated with academic performance.

**Table 13:** Hierarchical regressions predicting subject's average grade.

**Step**	**IV**	**Total *R*^2^**	***R*^2^ change**	**Standardized beta**	***t***
1	Backward digit-span	0.15	0.15	0.10	1.16
	CNT-attention control			0.06	0.67
	CNT-cognitive flexibility			−0.25	−2.89^**^
	Stroop-interference			0.25	2.87^**^
	SOC-problem solved in minimum move			0.14	1.75
	CGT-delay aversion			−0.03	−0.39
	CGT-risk adjustment			−0.09	−1.01

*CNT, Contingency Naming Test; CGT, Cambridge Gambling Task; SOC, Stockings of Cambridge*.

** *p < 0.01*.

**Table 14:** Hierarchical regressions predicting subject's Mathematics grade.

**Step**	**IV**	**Total *R*^2^**	***R*^2^change**	**Standardized beta**	***t***
1	Backward digit-span	0.10	0.10	0.033	0.95
	CNT-attention control			0.035	0.84
	CNT-cognitive flexibility			−0.25	−3.08^*^
	Stroop-interference			0.23	2.80^*^
	SOC-problem solved in minimum move			−0.05	−1.018
	CGT-delay aversion			0.70	1.87
	CGT-risk adjustment			−0.06	−0.49

*CNT, Contingency Naming Test; CGT, Cambridge Gambling Task; SOC, Stockings of Cambridge*.

* *p < 0.05*.

#### Psychological adjustment

A hierarchical regression analysis controlling general intellectual ability was used to examine which cool or hot EF measures best predict the five psychological domains of the SDQ. Only CGT risk adjustment (*B* = −0.264, *t* = −3.053, *p* < 0.05, *R*^2^ = 0.10) accounted for an additional 10% variance in the emotional problems scale (see Table 15). This suggests that CGT risk adjustment was the only EF factor associated with emotional problems. No cool EF tests associated with any other psychological domains of SDQ.

**Table 15:** Hierarchical regressions predicting participants' parent-observed emotional problems from strengths and difficulties questionnaire.

**Step**	**IV**	**Total *R*^2^**	***R*^2^ change**	**Standardized beta**	***t***
1	Backward digit-span	0.10	0.10	−0.08	−0.84
	CNT-attention control			0.01	0.14
	CNT-cognitive flexibility			−0.04	−0.43
	Stroop-interference			−0.03	−0.31
	CGT-delay aversion			0.03	0.36
	CGT-risk adjustment			−0.28	−3.09^**^
	SOC-problem solved in minimum move			−0.10	−1.11

*CNT, Contingency Naming Test; CGT, Cambridge Gambling Task; SOC, Stockings of Cambridge*.

** *p < 0.01*.

## Discussion

Like many other subtopics of cognition that have been pulled “in from the cold,” EF research has flourished since embracing the goal of integrating motivational and emotional processes into the traditional EF framework in an effort to understand behavior and achievement in full context. Although there exist some findings on the relationships between EF and important developmental outcomes, a number of research gaps have remained. The present study examined the development of cool and hot EF in a group of Chinese adolescents and investigated how they are related to two major developmental outcomes: academic performance and psychological adjustment. The CNT, Stroop test, SOC, and Backward Digit Span were chosen to investigate the attentional control, cognitive flexibility, inhibition, planning, and working memory domains of cool EF. In contrast, CGT risk adjustment and CGT delay aversion, both of which require the flexible appraisal of motivationally significant stimuli, were chosen to measure hot EF. Four major results were found: (1) cool and hot EFs showed different patterns of age-related growth in adolescence, such that cool EF exhibited an ascending curve while hot EF showed a bell-shaped curve; (2) internal correlations were found among cool EF tests, but no correlation was identified between cool and hot EF; (3) CNT cognitive flexibility and the Stroop test among cool EF measures were better predictors of academic performance; and (4) CGT risk adjustment of hot EF was a better predictor of emotional problems.

### Findings on cool executive function

Consistent with our hypothesis and findings of past research (e.g., Adleman et al., 2002; Hooper et al., 2004; Prencipe et al., 2011), the present findings suggest an ascending trend in all cool EF tests with increased age. Despite showing incremental progress overall, different developmental trends were observed among cool EF measures. The findings suggest a progressive trend for increased working memory, attentional control, cognitive flexibility, and inhibition through adolescence. *Post-hoc* analysis indicated that the magnitude of these improvements was relatively small in early adolescence (i.e., from age 12 to 13), but with a growth spurt occurring around 14 years (see Figures 1, 2, 3, and 5). Conversely, the development of planning as measured by the SOC appeared to be gradual from early to middle adolescence, with a later developmental spurt observed around age 16. Together, these findings echo previous research suggesting that attentional control, cognitive flexibility, working memory, and inhibition, which are considered to be fundamental to other facets of EF, precede and facilitate development of other higher-order EF facets, might display an earlier developmental trajectory than higher-order EF, namely, planning (Barkley, 1997; Carlson and Moses, 2001).

### Findings on hot executive function

In comparison to the accelerated maturation that has been found in cool EF, hot EF showed a bell-shaped development curve, with an upward slope from early adolescence and a peak in middle adolescence (ages 14 and 15), and finally, a downward slope from middle to late adolescence (age 17). In other words, the sensitivity to reward magnitude and choice impulsivity were highest in middle adolescence, perhaps suggesting a period of heightened risk-taking propensity (e.g., Steinberg, 2004). This result is consistent with past studies showing that the limbic and paralimbic brain areas, which regulate processing of rewards, social information, and emotions, becomes more sensitive and more easily aroused around middle adolescence (Giedd, 2004; Blakemore and Choudhury, 2006).

### Relationship between cool and hot executive function on developmental outcomes

#### Relations among cool and hot executive function tests

Among all EF tests, borderline to significant correlations were found among CNT attentional control and other cool tests including Backward Digit Span, and Stroop. These results not only provide additional evidence for the associations among cool EF tests, but might also imply that attentional control is a cognitive ability shared across all cool EF facets. In fact, attentional control, also known as executive attention, refers to an individual's capacity to choose what they pay attention to (Astle and Scerif, 2009; Sarter and Paolone, 2011), is closely related to other EF facets, such as working memory and inhibition (Mangun, 2012). In addition, although there were correlations among cool EF measures, no significant correlation was found between hot and cool EF measures. In other words, while cool EF seemed to be a reasonably coherent functional construct, hot EF was not.

#### Executive function in predicting developmental outcomes

As previously mentioned, research consistently shows a link between EF and academic outcomes in children, but findings on adolescents are scant. The present study confirmed this association and showed that two cool EF facets, inhibition and cognitive flexibility, significantly predict overall academic performance as well as Mathematics grades. This finding was consistent with earlier research finding that inhibitory skills are highly predictive of academic performance in children (Brock et al., 2009; Kim et al., 2013). In fact, most researchers agree that inhibition and cognitive flexibility are important in any problem-solving activity that requires inhibiting irrelevant or overlearned responses when generating response options (Zelazo et al., 1997), and that they are key abilities in learning. Nonetheless, it is important to note that performance in English and Chinese did not appear to be related to any of the cool EF measures. This might suggest that EF is less involved in some academic skills such as reading and writing (Clark et al., 2002) than skills that require more problem solving. Furthermore, in line with our hypothesis, the findings provide additional support that hot EF (i.e., CGT risk adjustment) might be particularly connected to emotional difficulties. In other words, adolescents with impaired or poor hot EF tend to be more impulsive and sensitive to risky activities (Johnson et al., 2009), further increasing their vulnerability to emotional challenges.

## Limitations and future directions

Although there were general agreement of the current findings with similar studies, a number of methodological limitations must be acknowledged. First, the sample size was relatively small, with around 136 participants. Future studies should include a larger sample size. Second, the current study assessed cool and hot EF using separate EF batteries. Future study should include a more comprehensive assessment battery that allows comparison between hot, affect-based vs. cool, deliberative-based EF in a single task. Examples such as the Columbia Card Task, which exists in two versions that separately trigger affective vs. deliberative decision-making processes in risky situations, might be ideal. Third, it is clear that bilingualism itself provides an advantage in EF through attention control (Bialystok, 2015). The current study examined a group of bilingual participants with Chinese as their native language and varying degree of proficiency in English. Generalization about the current data will be limited to this group of bilingual adolescents. Moreover, bilingual influence on EF has been extensively studied with children but is less studied with adolescents, future study should examine the associations between bilingual experiences and EF development in bilingual adolescents. Lastly, due to time constraints, a cross-sectional design was administered. The present study only made clear that specific cool and hot EF tests predict academic performance and emotional problems, and it could not demonstrate causal relationships. Future studies should adopt a longitudinal approach to provide valuable information on the relationships between lifespan development and cool and hot EF in adolescents. Nonetheless, the present study contributes several novel findings to the current literature. It is the first study to investigate the development of both hot and cool EF in the same sample of adolescents and to investigate whether and how these aspects of EF are related to important developmental outcomes. In addition, given the plastic nature of EF, especially in adolescence, the current findings may have practical implications for future EF identification and training.

## Author contributions

The author confirms being the sole contributor of this work and approved it for publication.

### Conflict of interest statement

The author declares that the research was conducted in the absence of any commercial or financial relationships that could be construed as a potential conflict of interest.
